# Outcomes for patients who are diagnosed with breast and endometrial cancer

**DOI:** 10.3892/ol.2013.1491

**Published:** 2013-07-25

**Authors:** TONYA M. MARTIN-DUNLAP, MITCHELL S. WACHTEL, JULIE A. MARGENTHALER

**Affiliations:** 1 Department of Surgery, Washington University, St. Louis, MO 63110, USA; 2 Department of Pathology, Texas Tech University Health Sciences Center, Lubbock, TX 79430, USA

**Keywords:** breast cancer, endometrial cancer, survival

## Abstract

The present study sought to determine the survival outcomes for women diagnosed with breast and endometrial cancer. Using SEER data, a population-based cohort study of women diagnosed with breast and endometrial cancer was conducted. Kaplan-Meier survival curves were created for disease-specific survival rates. A total of 2,027 women diagnosed with breast and endometrial cancer were identified. Of these, 1,296 (63.9%) developed breast cancer first and 731 (36.1%) developed endometrial cancer first. Regional lymph node involvement was significantly more common with a breast cancer diagnosis [522 (25.8%) women] compared with an endometrial cancer diagnosis [87 (4.3%) women] (P<0.05). Factors associated with decreased survival included a high tumor grade in endometrial cancer, nodal positivity and estrogen receptor-negative breast cancer (P<0.05 for each). There were 83 (4.1%) mortalities due to breast cancer, 63 (3.1%) mortalities due to endometrial cancer and 178 (8.8%) mortalities due to other causes (P<0.05). In conclusion, for women diagnosed with breast and endometrial cancer, the cumulative risk of mortality at five years following the second cancer diagnosis is nearly four times more likely to be due to breast cancer than endometrial cancer.

## Introduction

Breast cancer is the most common type of cancer diagnosed in women in the United States (US) (1). Cancer of the endometrium is the fourth most common cancer diagnosis in US women, following cancers of the lung and bronchus and the colon and rectum (1). Breast cancer is the second most common cause of cancer mortality in US women, following mortalities due to lung and bronchial cancers; endometrial cancers are eighth on the list of mortalities due to cancer in US women (1).

The American Cancer Society has estimated that there were 226,870 new cases of invasive breast cancer and 63,300 new cases of *in situ* breast cancer in the year 2012 (1). The lifetime risk for a diagnosis of breast cancer based on the 2006–2008 rates was reported at 12.29% (2). The Surveillance, Epidemiology and End Results (SEER) database reported the median age of diagnosis of breast cancer during 2004–2008 as 61 years old, while the median age of mortality due to breast cancer was 68 years old (2). The age-adjusted incidence rate during the same time frame was 124.0/100,000 women/year, while the age adjusted mortality rate was 23.5/100,000 women/year (2). The five-year relative survival for 2001–2007 in the SEER group was 89.1%. When adjusted by stage, the SEER reported a five-year relative survival of 98.6% for those with locally confined disease, 83.8% for those with regional lymph node disease and 23.3% for those with metastatic disease (2).

The American Cancer Society has estimated that there were 47,130 new cases of endometrial cancer in the year 2012 (1). The lifetime risk for a diagnosis of endometrial cancer based on the 2006–2008 rates was reported at 2.61% (3). The SEER database reported the median age of diagnosis of endometrial cancer during 2004–2008 as 61 years old, while the median age of mortality due to endometrial cancer was 72 years old (3). The age adjusted incidence rate during the same time frame was 23.9/100,000 women/year, while the age adjusted mortality rate was 4.2/100,000 women/year (3). The five-year relative survival for 2001–2007 in the SEER group was 81.8%. When adjusted by stage, the SEER reported a five-year relative survival of 95.8% for those with locally confined disease, 67.0% for those with regional lymph node disease and 15.9% for those with metastatic disease (3).

The overall survival outcomes of women who have been diagnosed with breast and endometrial cancer have not previously been reported in the literature. To that end, the present study investigated the survival data with regard to patients diagnosed with synchronous or metachronous breast and endometrial cancer, utilizing SEER data.

## Materials and methods

The present study was a retrospective, population-based cohort study of women with a primary diagnosis of invasive breast cancer plus a primary diagnosis of endometrial adenocarcinoma. The SEER program database was utilized to gather the study patients. The patients included in the study were diagnosed between January 1, 1988 and December 31, 2007. All study patients were recorded in the SEER database as not having evidence of distant metastases at the time of diagnosis. Additionally, all study patients had been followed up for at least one after the second cancer diagnosis was recorded.

The sequence of diagnosis of tumor type was recorded. The status at the end of the study was recorded as alive, breast cancer-related mortality, endometrial cancer-related mortality or mortality due to other causes. The histological grades were recorded as well-differentiated (grade I), moderately-differentiated (grade II), poorly-differentiated (grades III–IV) or unknown. The pathological lymph node status was recorded as negative, positive or unknown. The breast cancer receptor status for the estrogen receptor (ER) and the progesterone receptor (PR) was recorded as positive, negative or unknown. The age at the time of the second tumor diagnosis was recorded in years and the time between the first and second tumor diagnoses was recorded in months.

The endpoints for this study were breast cancer-specific mortality and endometrial cancer-specific mortality. These endpoints were recorded using the cause of mortality and the total completed months of follow-up noted in the SEER database.

A comparative risk regression analysis was used to analyze the risk of mortality secondary to breast cancer or endometrial cancer with regard to the tumor type at first diagnosis, the lymph node status, the histological differentiation of the two tumor types and the hormone receptor status. This analysis was dichotomized into an early follow-up period (<2.5 years) and a late follow-up period (2.5–5 years) to account for a survival crossover observed in the cumulative risk analysis. A cause-specific cumulative risk analysis was performed in the analysis of the risk of mortality with regard to the order of the tumor type diagnosis. All analyses utilized a null hypothesis rejection with a P-value of <0.05. All statistical analyses were performed using R version 2.13.0 of the cmprsk package (4).

## Results

### Patients and demographics

Using the SEER database, a total of 2,027 women who had a primary diagnosis of invasive breast cancer plus a primary diagnosis of endometrioid-type endometrial cancer were identified during the period of 1998–2007. Table I provides a summary of the patient and tumor characteristics that were utilized for the present study. During the study period, 1,296 women (63.9%) were identified with an initial cancer diagnosis of invasive breast cancer. The remaining 731 (36.1%) were women with an initial cancer diagnosis of endometrial cancer or those who had endometrial cancer diagnosed synchronously with their breast cancer. The median age at the time of the diagnosis of the second cancer was 68 years old. The median time measured between the initial diagnosis of cancer and the diagnosis of the second cancer type was 45 months. At the end of the study period, 1,703 women (84.0%) were still living, while 324 women (16%) had succumbed to various causes. The cause of mortality recorded in the SEER database was attributed to breast cancer in 83 women (4.1%), to endometrial cancer in 63 women (3.1%) and to other causes not associated with breast or endometrial cancer in 178 women (8.8%).

The tumor characteristics shown in Table I demonstrate that cancers of the endometrium were more likely to be of a lower histological grade at the time of diagnosis. Endometrial cancers were observed to be histologically well-differentiated in 913 of patients (45.0%), moderately-differentiated in 643 of patients (31.7%) and poorly-differentiated in 316 of patients (15.6%). This was compared with the findings in the breast tumors, which were histologically well-differentiated in 394 of patients (19.4%), moderately-differentiated in 813 of patients (40.1%) and poorly-differentiated in 641 of patients (31.6%). The histological grade could not be determined from the SEER database in 155 (7.6%) of patients with endometrial tumors and in 179 (8.8%) of patients with breast tumors. The lymph node status at the time of diagnosis was less likely to be known for the endometrial tumors, although when it was known, it was positive for disease in only 87 (4.3%) of patients and negative in 980 (48.3%) of patients. The lymph node disease burden in breast cancer was noted to be negative in 1,263 (62.3%) of patients and positive in 522 (25.8%) of patients. The hormone receptor status of the breast tumors revealed that the tumors were more likely to be positive rather than negative for ER and PR. The ER status was negative in 323 (15.9%) of tumors, positive in 1,364 (67.3%) of tumors and unidentifiable in 340 (16.8%) of tumors. The PR status was negative in 466 (23.0%) of tumors, positive in 1,178 (58.1%) of tumors and unidentifiable in 383 (18.9%) of tumors.

### Mortality risk analyses

**Figure 1:**
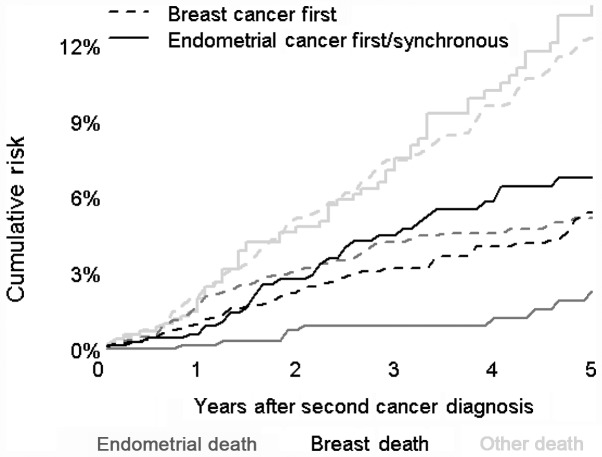
Cause-specific cumulative risk of mortality for 2,027 women diagnosed with breast and endometrial cancer.

The results of the analysis of the cause-specific cumulative risks of mortality are shown in Fig. 1. The greatest risk of mortality, independent of which tumor type was identified at the primary diagnosis, was attributed to factors other than breast or endometrial cancer. The risk of breast cancer being the cause of mortality was similar regardless of whether the patients were initially diagnosed with breast or endometrial cancer. The risk of mortality attributed to endometrial cancer was also similar to the risk of succumbing to breast cancer at the five-year time-point, if the tumor at the initial diagnosis was breast cancer. The risk of mortality attributed to endometrial cancer, if the tumor at the initial diagnosis was endometrial cancer, was lowest at the five-year time-point when compared with other causes.

**Figure 2:**
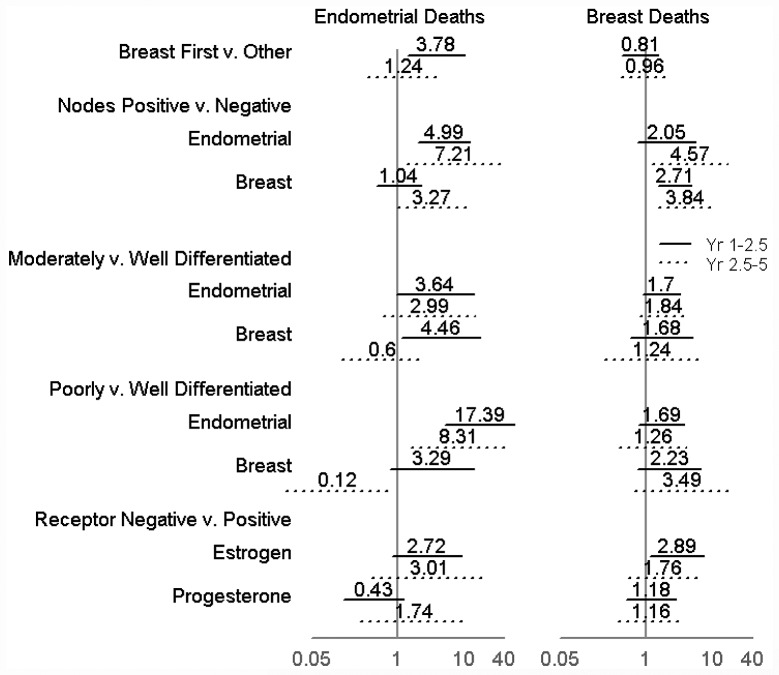
Regression analysis for risk of mortality for 2,027 women diagnosed with breast and endometrial cancer as adjusted for tumor-specific factors. The first half of the study period is represented by solid bars and the second half of the study is represented by broken bars. Hazard ratios (HR; 95% CI). Adjusted: age at second tumor, time between tumors. Not shown: comparisons with unknown.

The regression analyses of the comparative risk of endometrial cancer or breast cancer mortalities as associated with various factors are summarized in Fig. 2. The analysis was performed using two time periods in the study; the study time was divided at the 2.5-year mark. This was established due to the dichotomy of the endometrial cancer mortality cumulative risk lines observed in Fig. 1. The lines deviated from each other in the first half of the study, but became parallel in the second half.

### Prognostic factors

As expected, in the two halves of the study, positive lymph node disease was associated with an increased risk of mortality of the respective cancer type. The positive burden of breast cancer in the lymph nodes increased the risk of mortality from breast cancer in the first [Hazard ratio (HR), 2.71) and second half (HR, 3.84) of the study. The positive burden of endometrial cancer in the lymph nodes increased the risk of mortality from endometrial cancer in the first (HR, 4.99) and second half (HR, 7.21) of the study. The presence of lymph nodes with an endometrial cancer burden was also associated with a significant increase in the risk of mortality due to breast cancer in the second half of the study compared with the first half of the study (HR, 2.05 and HR, 4.57, respectively). The histological grade of breast cancer, when adjusted for other factors, did not have a significant association with breast cancer mortalities. Endometrial cancer mortalities did show an increased association with a poorly-differentiated tumor status upon histological examination when compared with the well-differentiated tumors. This effect was more significant in the first half of the study period compared with the second half (HR, 17.39 and 8.31, respectively). The risk of mortality from endometrial cancer was also observed to have a significant association with the differentiation level of the breast tumor. In the first half of the study, breast tumors with moderate or poor differentiation were associated with an increased risk of mortality from endometrial cancer (HR, 4.46 and 3.29, respectively). Conversely, in the second half of the study breast tumors with poor differentiation were associated with a decreased risk of mortality from endometrial cancer (HR, 0.12). The only association of significance with regard to hormone receptor status was identified in the first half of the study, whereby a negative ER status in a breast tumor was associated with an increased risk of mortality due to breast cancer (HR, 2.89).

## Discussion

Breast cancer is the most commonly diagnosed cancer in women in the US and endometrial cancer is the fourth most common cancer diagnosis (1). The present study investigated the impact of a synchronous or metachronous diagnosis of invasive breast and endometrial cancer on survival outcomes. This appears to be the first study of survival outcomes as impacted by these two types of cancer. The present study was an observational study of 2,027 women identified from the SEER database as having a diagnosis of both types of cancer. The results of this study may aid clinicians in treating patients diagnosed with both types of cancer.

The association of endometrial cancer following the treatment of a previously diagnosed breast cancer has been established in the literature, specifically with regard to the use of tamoxifen in the adjuvant treatment of breast cancer (5,6). The various types of endometrial cancer that develop in women during and after tamoxifen therapy have been previously studied in the literature (5,6). Women were observed to be more likely to develop a high-grade or high-risk type of endometrial cancer (type II) when the diagnosis was established following the cessation of tamoxifen. Bland *et al*(7) noted this difference after a six-month time frame from the completion of therapy and Ferguson *et al*(8) noted it after a 12-month period between tamoxifen therapy discontinuation and endometrial cancer diagnosis. It has also been observed that there is an increased risk of these high-risk subtypes of endometrial cancer in women who complete the standard five-year course of tamoxifen therapy compared with those are administered it for <5 years (7). The present study specifically investigated the endometrioid variant of endometrial cancer, which is classified as a type I endometrial tumor in the majority of cases, although if it is of a high histological grade it may be classified as a type II tumor. We were unable to discern with certainty whether any cases of endometrial cancer in the present patients were due to tamoxifen therapy, as this variable was not recorded in the SEER dataset. There were 1,296 (63.9%) women who were diagnosed with breast cancer first and 67.3% of the breast tumors in the study were ER-positive. There was a median of 45 months and an interquartile range of 17–81 months between the diagnoses of the first and second tumors. We would infer from this data that a significant percentage of these patients were likely offered endocrine therapy as adjuvant treatment for their breast cancer, but we are unable to determine whether tamoxifen was utilized, versus an aromatase inhibitor, or whether there was a causal relationship with the patients’ subsequent endometrial cancer.

In the present study, the risk of mortality due to other causes was greater than the risk of mortality from either breast or endometrial cancer. This finding of ‘other cause’ mortality has been documented in previous literature for breast cancer, but the data is unclear on this matter for endometrial cancer (9). In the present analysis, it was shown that the risk of mortality from breast cancer was similar regardless of which tumor type was diagnosed initially. By contrast, the risk of mortality from endometrial cancer was markedly lower if the initial diagnosis was endometrial cancer. This may also be related to the fact that patients whose first diagnosis was breast cancer would likely have received tamoxifen and subsequently were at risk of developing a higher grade of endometrial cancer, as opposed to those whose initial diagnosis of endometrial cancer was more likely to be of a lower tumor grade (10,11).

There were a number of unexpected outcomes from the present analysis. As expected, the patients with the higher histological grades of endometrial cancer were more likely to succumb to endometrial cancer. However, this effect was not observed in the analyses of histological grades and breast cancer-specific survival. Although the histological grade of breast tumors has been shown to be correlated with a poorer prognosis in previous studies (12–14), the present study did not observe any significant effect on the risk of mortality from breast cancer based on the increasing histological grade. There was an unexpected effect of the breast tumor histological grade on the risk of mortality from endometrial cancer. In the present study, patients who had high-grade breast tumors were at an increased risk of mortality due to endometrial cancers in the first half of the study. This effect was not observed in the second half of the study. The clinical significance of this finding is unclear. It may be a reflection of the shorter interval between the two cancer diagnoses and the more aggressive biology of the endometrial cancer.

As expected, as lymph node burden increases for a specific cancer, there is a concomitantly increased risk of mortality from that specific disease. An unexpected finding was observed in the later stage of the study, where the lymph node burden of endometrial cancer showed significance in an increased association with mortality due to breast cancer. There was also an increased risk of mortality due to endometrial cancer with a positive lymph node burden of breast cancer in the second half of the study, although this association was not at a statistically significant level. Although these correlations between lymph node disease and mortality due to the opposing cancer type were of statistical interest in the present analyses, it is unclear whether there is a clinical link between the two histologies that would result in this finding.

There are a number of limitations to the present study that are derived from its nature as a retrospective cohort study. The primary outcome that was assessed was cancer-specific mortality, but the comparison only included patients with a diagnosis of both cancer types. A helpful addition would be the comparison of this group with patients with a diagnosis of breast or endometrial cancer only. There are also limitations associated with the use of the SEER database and the information available for analysis. The addition of information with regard to the comorbidities, the margin status of tumor resections and the adjuvant treatments are important variables that were not available in the present analysis of this specific group of patients. The majority of patients did not have lymph node disease and there was a large volume of patients with unknown lymph node status in the endometrial cancer group. The results of the analysis may be different if a group of patients with a larger burden of disease at diagnosis was examined. Despite these limitations, the SEER database is a large population database that is used frequently in epidemiological studies (15).

The present study provides the first mortality analysis of patients with either synchronous or metachronous breast and endometrial cancer, two commonly diagnosed cancers among women in the US. These findings should be considered when clinicians enter discussions concerning prognoses with patients of similar standing. It is important to encourage patients to continue surveillance for a second type of cancer even after they have been diagnosed with a primary type of cancer. It is equally important for clinicians to continue screening practices for other cancers for patients who have been treated for another cancer diagnosis. For example, women who have been diagnosed with endometrial cancer should be encouraged to continue to undergo annual screening mammography. Furthermore, it is of particular importance for clinicians to educate patients who have been treated with adjuvant tamoxifen for breast cancer on the signs and symptoms of endometrial cancer and the necessity of reporting these signs and symptoms to their physician in a timely manner so that diagnostic interventions may be utilized.

## Figures and Tables

**Table I:** Summary of characteristics of interest for 2,027 women diagnosed with breast and endometrial carcinoma.

Characteristic	Value
First tumor diagnosis, n (%)	
Breast cancer	1296 (63.9)
Endometrial cancer/synchronous	731 (36.1)
Status at end of study, n (%)	
Alive	1703 (84.0)
Breast mortality	83 (4.1)
Endometrial mortality	63 (3.1)
Other mortality	178 (8.8)
Endometrium histological grade, n (%)	
Well-differentiated (SEER grade I)	913 (45.0)
Moderately-differentiated (SEER grade II)	643 (31.7)
Poorly-differentiated (SEER grades III–IV)	316 (15.6)
Unknown	155 (7.6)
Breast histological grade, n (%)	
Well-differentiated (SEER grade I)	394 (19.4)
Moderately-differentiated (SEER grade II)	813 (40.1)
Poorly-differentiated (SEER grades III–IV)	641 (31.6)
Unknown	179 (8.8)
Endometrium lymph node status, n (%)	
Negative	980 (48.3)
Positive	87 (4.3)
Unknown	960 (47.4)
Breast lymph node status, n (%)	
Negative	1263 (62.3)
Positive	522 (25.8)
Unknown	242 (11.9)
Breast ER status, n (%)	
Negative	323 (15.9)
Positive	1364 (67.3)
Unknown	340 (16.8)
Breast PR status, n (%)	
Negative	466 (23.0)
Positive	1178 (58.1)
Unknown	383 (18.9)
Median age at second tumor diagnosis, years (interquartile range)	68 (60–76)
Median time between first and second tumors, months (interquartile range)	45 (17–81)

SEER, Surveillance, Epidemiology and End Results; ER, estrogen receptor; PR, progesterone receptor.
